# Subjective Cognitive Impairment Cohort (SCIENCe): study design and first results

**DOI:** 10.1186/s13195-018-0390-y

**Published:** 2018-08-07

**Authors:** Rosalinde E. R. Slot, Sander C. J. Verfaillie, Jozefien M. Overbeek, Tessa Timmers, Linda M. P. Wesselman, Charlotte E. Teunissen, Annemiek Dols, Femke H. Bouwman, Niels D. Prins, Frederik Barkhof, Adriaan A. Lammertsma, Bart N. M. Van Berckel, Philip Scheltens, Sietske A. M. Sikkes, Wiesje M. Van der Flier

**Affiliations:** 1 Alzheimer Center and Department of Neurology, Amsterdam Neuroscience, Amsterdam University Medical Centers, Vrije Universiteit, De Boelelaan 1118, 1081 HZ Amsterdam, The Netherlands; 2 Neurochemistry Laboratory, Department of Clinical Chemistry, Amsterdam University Medical Centers, Vrije Universiteit, Amsterdam, The Netherlands; 3 Department of Old Age Psychiatry, GGZ InGeest, Amsterdam University Medical Centers, Vrije Universiteit, Amsterdam, The Netherlands; 4 Department of Radiology and Nuclear Medicine, VU University Medical CenterAmsterdam University Medical Centers, Vrije Universiteit, Amsterdam, The Netherlands; 5 Institutes of Neurology and Healthcare Engineering, UCL, London, UK; 6 Department of Epidemiology and Biostatistics, Amsterdam University Medical Centers, Vrije Universiteit, Amsterdam, The Netherlands

**Keywords:** Subjective cognitive decline, Preclinical Alzheimer’s disease, Subthreshold psychiatry, SCD-plus criteria, Study design

## Abstract

**Background:**

We aimed to describe the Subjective Cognitive Impairment Cohort (SCIENCe) study design, to cross-sectionally describe participant characteristics, and to evaluate the SCD-plus criteria.

**Methods:**

The SCIENCe is a prospective cohort study of subjective cognitive decline (SCD) patients. Participants undergo extensive assessment, including cerebrospinal fluid collection and optional amyloid positron emission tomography scan, with annual follow-up. The primary outcome measure is clinical progression.

**Results:**

Cross-sectional evaluation of the first 151 participants (age 64 ± 8, 44% female, Mini-Mental State Examination 29 ± 2) showed that 28 (25%) had preclinical Alzheimer’s disease (AD) (amyloid status available *n* = 114 (75%)), 58 (38%) had subthreshold psychiatry, and 65 (43%) had neither. More severe subjective complaints were associated with worse objective performance. The SCD-plus criteria age ≥ 60 (OR 7.7 (95% CI 1.7–38.9)) and apolipoprotein E (genotype) e4 (OR 4.8 (95% CI 1.6–15.0)) were associated with preclinical AD.

**Conclusions:**

The SCIENCe study confirms that SCD is a heterogeneous group, with preclinical AD and subthreshold psychiatric features. We found a number of SCD-plus criteria to be associated with preclinical AD. Further inclusion and follow-up will address important questions related to SCD.

**Electronic supplementary material:**

The online version of this article (10.1186/s13195-018-0390-y) contains supplementary material, which is available to authorized users.

## Background

Alzheimer’s disease (AD) develops gradually, and the first pathophysiological changes occur decades before a diagnosis of dementia [1, 2]. Research interest is shifting to increasingly earlier stages, as the origin of AD and keys to treatment probably lie in the prevention of progression to fully fledged disease. Preclinical AD is defined as an asymptomatic stage of AD, in which AD biomarkers are aberrant but clinical symptoms of objective cognitive decline are not present [3]. Subjective cognitive decline (SCD) refers to the experience of cognitive decline, without formal deficits on neuropsychological testing, or any other neurological or psychiatric diagnosis explaining cognitive complaints [4]. The subjective experience of cognitive decline has been suggested to be one of the first symptoms of AD, and patients with SCD have an increased risk of progression to MCI or dementia, especially when complaints are reported by both patient and informant [5–10]. In cognitively normal individuals with SCD, biomarkers of AD can already be aberrant, such as low CSF amyoid-beta_1–42_, increased amyloid deposition on PET scans, and thinner medial temporal cortex [11–14]. However, the sequence of neurodegenerative changes eventually leading to AD may vary amongst individuals, and where to place SCD in these pathological sequences remains to be elucidated.

It is difficult to clinically identify preclinical AD in cognitively healthy individuals experiencing memory complaints. To increase the likelihood of preclinical AD in individuals with SCD, the SCD-I Working Group has proposed the SCD-plus criteria [4]. These criteria include biomarkers such as APOE e4 carriership, but also patient specific features such as predominant self-perceived memory decline and feeling of worse memory performance than others of the same age. The SCD-plus criteria have been proposed to facilitate harmonizing SCD research, but they have not yet been prospectively validated.

Even though individuals with SCD on average have an increased risk of AD, most individuals with SCD do not harbor Alzheimer pathology. Alternative potential explanations for the experience of memory complaints in cognitive healthy individuals include subthreshold symptoms of affective disorders, personality features, lifestyle factors, or systemic illnesses [15–17]. To evaluate the contribution of different factors related to SCD we have set up the memory clinic-based Subjective Cognitive Impairment Cohort (SCIENCe). In this ongoing cohort study we investigate individuals with SCD, without major psychiatric of neurological disorders. Here, we aimed to describe the SCIENCe study design, to cross-sectionally evaluate participants characteristics and factors related to cognitive complaints, and to evaluate the recently defined SCD-plus criteria as indicators of preclinical AD.

## Methods

### Study design and work-up

The Subjective Cognitive Impairment Cohort (SCIENCe) is a prospective cohort study including consecutive patients with SCD presenting at the Alzheimer Center of the VU University Medical Center Amsterdam. Here, we extensively describe the study design of the ongoing SCIENCe study. In addition, we report results based on a selection of cross-sectional data of the first 151 SCIENCe participants.

Inclusion criteria for the SCIENCe are a diagnosis of SCD (i.e., cognitive complaints and normal cognition) and age ≥ 45 years. Exclusion criteria are MCI, dementia, major psychiatric disorder (i.e., current depression, personality disorders, schizophrenia), neurological diseases known to cause memory complaints (i.e., Parkinson’s disease, epilepsy), HIV, abuse of alcohol or other substances, and language barrier.

All participants have been referred to the memory clinic by their general practitioner, and a neurologist or geriatrician in the case of a second opinion for evaluation of cognitive complaints. They receive standardized dementia screening at the memory clinic, including an interview with a neurologist, physical and neurological examination, neuropsychological assessment, as well as routine analyses of blood, CSF, and brain magnetic resonance imaging (MRI). After the standardized dementia screening, diagnoses are made in a multidisciplinary consensus meeting. Patients receive a label of SCD when cognitive functioning is normal and when there is no diagnosis of MCI, dementia, or any other disease known to cause memory complaints [18]. When subtle symptoms of an underlying psychiatric diagnosis, such as depression, are suspected, patients are evaluated by an experienced psychiatrist to exclude possible formal psychiatric diagnoses as the cause of cognitive complaints.

**Fig. 1:**
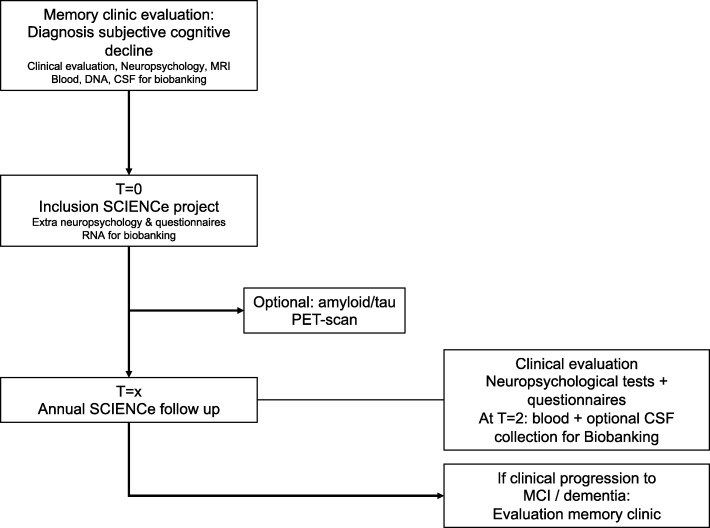
SCIENCe work-up. SCIENCe work-up at baseline and annual follow-up study visits. Primary outcome is clinical progression to MCI or dementia. CSF cerebrospinal fluid, MCI mild cognitive impairment, MRI magnetic resonance imaging, PET positron emission tomography, SCIENCe Subjective Cognitive Impairment Cohort

Eligible patients with SCD are invited to participate in the SCIENCe. After inclusion in the SCIENCe, participants are invited for additional baseline assessments, which are described in detail in the following. After completion of baseline assessment, patients are invited for an annual follow-up visit consisting of clinical evaluation, extensive neuropsychological assessment, and questionnaires. At each follow-up visit, diagnoses are reevaluated under supervision of a neurologist. Main outcome measures are clinical progression to MCI or dementia and decline in cognitive functioning. If patients progress to MCI or dementia they are offered the possibility to return to a routine memory clinic follow-up. The SCIENCe work-up is visualized in Fig. 1.

The local medical ethics committee of the VU University Medical Center approved the study and all patients provide written informed consent for the use of their clinical data and biomaterial in research. All research is conducted in accordance with the Helsinki Declaration of 1975.

**Fig. 2:**
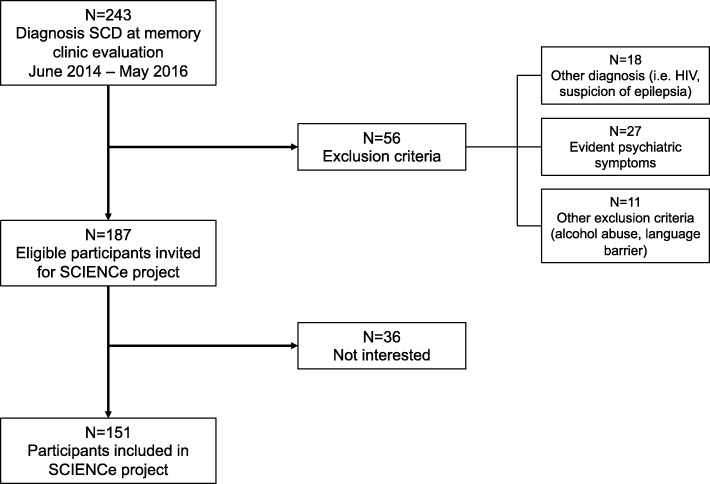
Flow chart of study inclusion. Flow-chart of inclusion of SCIENCe participants evaluated in current report (*n* = 151). Further inclusion and follow-up currently ongoing. SCD subjective cognitive decline, SCIENCe Subjective Cognitive Impairment Cohort

SCIENCe inclusion started in June 2014. In the first 2 years, 243 consecutive individuals aged 45 years or older received a diagnosis of SCD, of which 56 were not eligible for participation and 36 individuals were not interested in participation (Fig. 2). This led to inclusion of 151 individuals in the SCIENCe until the start of data analysis for the current report. In this cross-sectional report of SCIENCE baseline findings, we evaluate these first 151 participants. Further inclusion in the SCIENCe and follow-up of participants is currently ongoing.

### Questionnaires

Additional file 1: Table S1 provides a detailed overview of the questionnaires used to evaluate SCD, mental health, instrumental activities of daily living, and lifestyle (i.e., dietary intake, and physical and cognitive activity).

#### Subjective cognitive decline

We use the Dutch translation of the Cognitive Change Index—self (CCI-S) and Cognitive Change Index—informant report (CCI-I) (20 questions, range 0–80) to assess cognitive function compared to 5 years ago [13]. Higher scores reflect worse subjective cognitive function. The CCI cutoff value for significant cognitive complaints is set at 16/80 [19]. In addition, we use the Subjective Cognitive Functioning (SCF) questionnaire (four questions, range − 12 to + 12) to assess self-experienced cognitive decline over a 1-year time period [20]. A SCF score below 0 represents decline.

#### Psychiatric symptoms

We use the following questionnaires to evaluate psychiatric symptoms: depressive symptoms, CES-D [21]; anxiety, HADS-A [22]; neuroticism, NPV neuroticism subscale [23, 24], low mastery, Pearlin Mastery scale [25]; distress and somatization (defined as nonspecific physical complaints), 4-DKL distress and somatization subscales [23]; and quality of life, EuroQol [26]. For all psychiatric and quality-of-life questionnaires, higher scores reflect worse performance. See Additional file 1: Table S1 for cutoff values of the questionnaires.

### Neuropsychological evaluation

All participants received a comprehensive standardized neuropsychological assessment at the regular memory clinic evaluation [18]. As part of the SCIENCe baseline investigation we perform an additional neuropsychological assessment (median time between assessments 37 days) evaluating cognitive domains: memory, language, attention, executive and visuospatial functioning, with a special emphasis on memory; see Additional file 1: Table S1 for an overview of the complete SCIENCe test battery. This test battery is repeated at follow up.

In this paper we report on a subset of the neuropsychological assessment. We used the MMSE to assess global cognition [27]. For the memory domain we used the Dutch version of the Rey Auditory Verbal Learning Test (RAVLT)—direct recall (five trials summed) and delayed recall and cued recall (both > 20 min) [28]. We used Trail Making Test (TMT) A to evaluate attention, and TMT B to evaluate executive functioning [29]. To evaluate language functioning we used categorical animal fluency.

### Magnetic resonance imaging

Structural MRI is acquired during the diagnostic visit to the memory clinic using a MR750 (General Electric, Milwaukee, WI, USA), Philips PET/MR (Philips Medical Systems, Best, the Netherlands), or Toshiba Titan (Toshiba Medical Systems, Otawara, Japan). The MRI protocol includes isotropic 3D T1-weighted and Fluid Attenuated Inversion Recovery (FLAIR) T2-weighted, and Susceptibility Weighted Imaging (SWI). T1-weighted images are used to estimate hippocampal and normalized brain volumes (NBV) using FIRST and SIENAX with optimized settings (FMRIB software library v5, Oxford, UK) [30, 31] derived from a tissue-type segmentation, using optimized parameter settings and a scaling factor to normalize for skull size [31]. All registrations are visually inspected for artifacts. All images are read by a neuroradiologist in a standardized fashion. The severity of white-matter hyperintensities (WMHs) using the Fazekas scale is determined on the FLAIR sequence (possible range 0–3), and dichotomized into absent (0–1) or present (2–3). Lacunes are defined as deep lesions (3–15 mm) with CSF-like signal on all sequences. Lacunes are scored as absent or present (≥ 1 lacune). Microbleeds are defined as small dot-like hypointense lesions on T2-weighted MRI. Microbleed count is dichotomized into absent or present (≥ 1 microbleed). Here, we present baseline normalized brain volume, bilateral hippocampal volume, WMHs, lacunes, and microbleeds. MRI data within 1 year from SCIENCe inclusion were available for 116 (77%) participants.

### Biomaterial for biobanking

Blood (serum and plasma), DNA, and CSF are obtained and stored in our biobank at the department of Clinical Chemistry of the VU University Medical Center Amsterdam, according to international consensus standard operation procedures [32, 33].

#### Blood and DNA

Venous blood (2–6 ml clotted blood for serum and 6 ml EDTA blood for plasma) is processed and stored according to international consensus standard operation procedures. EDTA whole blood (2–4 ml) is collected for DNA extraction. After collection, plasma and serum samples are centrifuged at room temperature at 2000 × *g* (minimum 1800 × *g*, maximum 2200 × *g*), aliquoted into 0.5-ml vials, and stored at − 80 °C.

#### RNA

After SCIENCe inclusion, one PAXgene Blood RNA tube (PreAnalytiX; Qiagen, Venlo, the Netherlands) is collected and without aliquoting stored in the biobank at − 80 °C.

#### CSF

CSF is collected from nonfasted subjects. CSF is obtained by lumbar puncture between the L3/L4 or L4/L5 intervertebral space by a 25-gauge needle and collected in polypropylene tubes.

After collection, CSF and plasma samples are centrifuged at room temperature at 2000 × *g* (minimum 1800 × *g*, maximum 2200 × *g*), aliquotted into 0.5-ml vials, and stored at − 80 °C. A maximum of 2 h is allowed between collection and freezing [32, 33].

### *APOE* genotyping

*APOE* genotyping is performed after automated genomic DNA isolation from 2–4 ml EDTA blood. It is subjected to PCR, checked for size and quantity using a QIAxcel DNA Fast Analysis kit (Qiagen), and sequenced using Sanger sequencing on an ABI130XL. Here, *APOE* status was available for 144 (95%) individuals. Subjects with one or two ε4 alleles were classified as *APOE* e4 carriers.

### Cerebrospinal fluid markers

From the total amount of collected CSF at memory clinic visit, 2.5 ml is used for routine analyses, including leukocyte count, erythrocyte count, glucose concentration, and total amount of protein, and frozen at − 20 °C until further analysis of Alzheimer biomarkers. Amyloid-beta_1–42_ (Aβ42), tau, and tau phosphorylated threonine 181 (ptau) levels are measured using ELISA (Innogenetics-Fujirebio, Ghent, Belgium) at the Neurochemistry Laboratory [34]. Our center cutoff value for CSF Aβ42 indicating AD pathology is < 640 μg/L [35].

### Positron emission tomography scans

All participants are invited to participate additionally in an amyloid PET study. Patients are scanned with either [^18^F]florbetapir (Amyvid) or [^18^F]florbetaben (Neuraceg) radiotracer. Before scanning, one cannula is inserted for tracer infusion. For florbetapir, 90-min dynamic PET emission scans (PET/CT Ingenuity TF or Gemini TF; Philips Medical Systems) are acquired immediately following bolus injection of approximately 370 MBq [^18^F]florbetapir. For florbetaben, 20-min static PET emission scans (PET/MR; Philips Medical Systems) are acquired 90 min after a bolus injection of approximately 250 MBq [^18^F]florbetaben. All PET scans are visually read by a nuclear medicine physician. For the current manuscript, PET scans were available for 105/151 (69%) participants.

### Amyloid status

Information on amyloid status was available for 114 (75%) participants (PET only *n* = 38 (25% of total), CSF only *n* = 9 (6%), CSF and PET *n* = 67 (44%)). Amyloid status could be determined if: CSF and/or amyloid PET was performed within 1 year of baseline visit; or if repeated amyloid measurements were concordant before and after baseline (i.e., both negative or both positive). There were seven cases with discordant PET/CSF results. In all seven cases, CSF Aβ42 was above the cutoff value of 640 μg/L (range 645–881 μg/L), but amyloid PET was positive; we considered these cases as amyloid positive.

### Categorization of participants according to concomitant symptoms

In this cross-sectional report of SCIENCE baseline findings, we categorized SCIENCe participants into categories based on the presence of preclinical AD and/or subthreshold psychiatry, as potential factors associated with SCD [4].

#### Preclinical AD

Amyloid-positive individuals based on PET and/or CSF amyloid (see Amyloid status) were classified as preclinical AD.

#### Subthreshold psychiatry

Individuals with one or more questionnaires indicative of subthreshold symptoms of depression, anxiety, neuroticism, low mastery, distress, or somatization were classified as subthreshold psychiatry (see Additional file 1: Table S1 for an overview of questionnaires and cutoff values used). Fulfillment of clinical criteria for a formal psychiatric diagnosis was an exclusion criterion for the SCIENCe, hence psychiatric symptoms measured with the questionnaires were subthreshold. When participants were amyloid positive but also had subthreshold psychiatric symptoms they were classified in the preclinical AD group. Amyloid status was not available in the subthreshold psychiatry category for 21 of 58 cases (36%).

#### Undetermined

When participants were neither amyloid positive or there was no indication of subthreshold psychiatric symptoms, they were classified in the undetermined category. Amyloid status was not available in the undetermined category for 16 of 65 patients (25%).

### SCD-plus criteria

The SCD-plus criteria refer to specific features of SCD associated with an increased likelihood of preclinical AD [4]. The SCD-plus criteria are: (1) *subjective decline in memory, rather than other domains of cognition* (in our study defined as ‘memory decline present’ as evaluated in the SCF questionnaire); (2) *onset of SCD within the last 5 years*; (3) *age at onset of SCD ≥ 60 years*; (4) *concerns (worries) associated with SCD*; (5) *feeling of worse performance than peers* (here operationalized with a specific question in the CCI questionnaire); (6) *confirmation of perceived cognitive decline by an informant* (here operationalized as a CCI informant report score above cutoff value of significant symptoms (> 16)); and (7) *APOE e4 carriership*. We evaluated the SCD-plus criteria with the exception of criterion *worries associated with SCD* (4), which we considered present in all participants since they all visited our memory clinic because of cognitive complaints.

### Statistical analyses

Data were analyzed using IBM SPSS Statistics, version 22 (IBM, Armonk, NY, USA). We assessed baseline features of the study population and evaluated differences between participant categories (preclinical AD, subthreshold psychiatry, or undetermined), using chi-squared tests or ANOVA, adjusted for age and gender, as appropriate, followed by post-hoc analyses. We used univariate linear regression analyses to assess associations between cognitive complaints (CCI-S, CCI-I, and SCF) and neuropsychological test scores, adjusted for age and gender.

Furthermore, we evaluated the prevalence of the SCD-plus criteria in participants with available amyloid status. Subsequently, we used logistic regression to investigate the associations of SCD-plus criteria with the risk of preclinical AD. First, we performed univariate models with each SCD-plus criterion separately (model 1). Then, we constructed model 2 as a multivariate model with backward stepwise selection with the six available SCD-plus criteria. We considered *p* < 0.05 significant.

## Results

### Baseline demographics

At baseline the first 151 SCIENCe participants were on average 64 ± 8 years old (range 45–84 years), and 67 (44%) were female (Table 1). Participants received on average 12 ± 3 years of education, and 76 (54%) had a family history of dementia. Fifty-five participants (38%) were APOE e4 positive (APOE e4 status available for *n* = 144 (95%)).

**Table 1:** Demographic features of the study population

		*n*	Total group (*N* = 151)		Preclinical AD (*n* = 28)	Subthreshold psychiatry (*n* = 58)	Undetermined (*n* = 65)	*p*
Demographics	Age	151	64 ± 8		69 ± 7^b^	62 ± 8^a^	64 ± 8^a^	0.002
	Gender, female	151	67 (44)		11 (39)	27 (47)	29 (45)	NS
	Education (years)	148	12 ± 3		13 ± 3	12 ± 3	12 ± 2	NS
	Family history dementia	140	76 (54)		18 (75)^b^	20 (36)^a^	38 (62)^b^	0.002
	*APOE* e4 carrier	144	55 (38)		17 (65)^b^	17 (30)^a^	21 (34)^a^	0.007
				*n* above cutoff value				
Subjective cognitive decline	SCF (1-year change) self-report	150	−1.65 ± 2.98	104 (69)	−2.0 ± 2.3	−2.4 ± 3.2	−0.8 ± 2.9^b^	0.004
	CCI (5-year change) self-report^c^	148	21.8 ± 14.3	89 (60)	21.4 ± 13.2^b^	27.8 ± 15.2^a^	16.7 ± 11.8^b^	0.000
	CCI (5-year change) informant^c^	127	19.4 ± 17.1	62 (49)	19.8 ± 13.9^b^	26.4 ± 19.1^a^	13.8 ± 14.8^b^	0.000
Mental health questionnaires	Quality of Life	149	76 ± 15		79 ± 12	71 ± 16	80 ± 15^b^	0.012
	Depressive symptoms^c^	150	8.3 ± 6.4	17 (11)	7.0 ± 4.6	12.1 ± 7.3	5.5 ± 4.1	0.000
	Anxiety^c^	150	4.0 ± 2.9	13 (13)	4.1 ± 2.6	5.6 ± 3.1	2.4 ± 1.9	0.000
	Distress^c^	150	6.7 ± 5.9	34 (22)	4.6 ± 4.6	11.1 ± 6.3	3.6 ± 2.9	0.000
	Somatization^c^	151	6.3 ± 5.3	31 (21)	4.6 ± 3.7	10.3 ± 5.7	3.5 ± 2.8	0.000
	Neuroticism^c^	145	6.6 ± 5.5		5.2 ± 3.8	10.1 ± 6.4	4.0 ± 2.8	0.000
	Low mastery^c^	140	10.5 ± 3.9		10.0 ± 3.0	12.8 ± 4.0	8.5 ± 2.7	0.000
Cognition	MMSE	151	28.6 ± 1.2		28.4 ± 1.3	28.5 ± 1.2	28.9 ± 1.2	0.031
Memory domain	RAVLT immediate recall	149	44.3 ± 9.0		43.4 ± 8.7	44.0 ± 9.0	44.6 ± 9.2	NS
	RAVLT delayed recall	149	9.0 ± 2.9		8.5 ± 2.9	9.1 ± 3.0	9.2 ± 2.9	NS
	RAVLT cued recall	149	28.7 ± 1.6		28.7 ± 1.5	28.5 ± 2.3	28.8 ± 1.4	NS
Attention	TMT A^c^	148	34.4 ± 12.8		33.4 ± 12.2^b^	37.5 ± 14.9^a^	32.1 ± 10.5^b^	0.014
Executive functioning	TMT B^c^	147	82.1 ± 33.2		79.5 ± 28.9	89.8 ± 41.0	76.2 ± 25.8^b^	0.058
Language	Animal fluency	147	23.2 ± 5.2		22.8 ± 4.9	23.1 ± 5.1	23.5 ± 5.6	NS
MRI	Normalized brain volume (ml)	116	1399 ± 79		1366 ± 75	1407 ± 81	1406 ± 79	NS
	Bilateral hippocampal volume (ml)	116	9.9 ± 1.3		10.0 ± 1.4	9.9 ± 1.3	9.8 ± 1.1	NS
	White-matter hyperintensities (present)	116	10 (9)		2 (9)	2 (4)	6 (13)	NS
	Lacunes (> 0)	115	3 (3)		1 (4)	2 (5)	0 (0)	NS
	Microbleeds present (> 0)	112	19 (17)		7 (30)	4 (10)	8 (17)	NS

Unadjusted results presented as mean ± standard deviation or *n* (%). Differences between groups assessed using age, gender, and education-adjusted analysis of variance or chi-squared tests

*AD* Alzheimer’s disease, *APOE* apolipoprotein E genotype, *CCI* cognitive change index, *MMSE* Mini-Mental State Examination, *MRI* magnetic resonance imaging, *NS* not significant, *RAVLT* Rey Auditory Verbal Learning Test, *SCF* subjective cognitive functioning, *TMT* Trail Making Test

^a^*p* < 0.05 difference with preclinical AD

^b^*p* < 0.05 difference with subthreshold psychiatry

^c^Higher scores reflect worse performance or more symptoms

### Self-report of SCD

We cross-sectionally assessed report of subjective cognitive functioning compared to 1 year ago (SCF self-report) and 5 years ago (CCI; both self-report and informant report; Table 1). Over the preceding 5-year time period 146 (97%) participants reported cognitive decline (CCI-S), of which 89 (60%) reported substantial decline. Over a 1-year time period (SCF), 104 (69%) participants reported substantial cognitive decline. Adjusted for age, gender, and education, higher CCI-S was associated with worse SCF (standardized β = − 0.40, *p* < 0.001), and CCI-S was also associated with CCI-I (sβ = 0.48, *p* < 0.001; Table 2). In addition, we found that higher self-report of subjective cognitive functioning (CCI-S and SCF) was associated with worse quality of life (sβ = − 0.34; sβ = 0.25; both *p* < 0.05). Furthermore, higher CCI (both self and informant) were associated with worse performance on cognitive tests (Table 2), while there were no associations between SCF and objective measures of cognition.

**Table 2:** Associations between subjective and objective cognitive measures

	SCF	CCI-self	CCI-informant
CCI-self^a^	− 0.39**		
CCI-informant^a^	− 0.19*	0.49**	
EuroQol	0.25*	− 0.33**	− 0.15
MMSE	0.14	− 0.30**	− 0.10
RAVLT immediate recall	0.01	− 0.21*	− 0.15
RAVLT delayed recall	0.03	− 0.16	− 0.04
RAVLT cued recall	− 0.12	− 0.23*	− 0.17*
TMT A^a^	− 0.06	0.12	0.17
TMT B^a^	− 0.17	0.23*	0.26*
Animal fluency	0.15	− 0.15	− 0.14

Associations presented as standardized β, adjusted for age, gender, and education

*SCF* subjective cognitive functioning (lower scores indicate more complaints), *CCI* cognitive change index (higher scores indicate more complaints), *MMSE* Mini-Mental State Examination, *RAVLT* Rey Auditory Verbal Learning Test, *TMT* Trail Making Test

*p < 0.05

**p < 0.001

^a^Higher scores reflect worse cognitive performance

### SCD groups

**Fig. 3:**
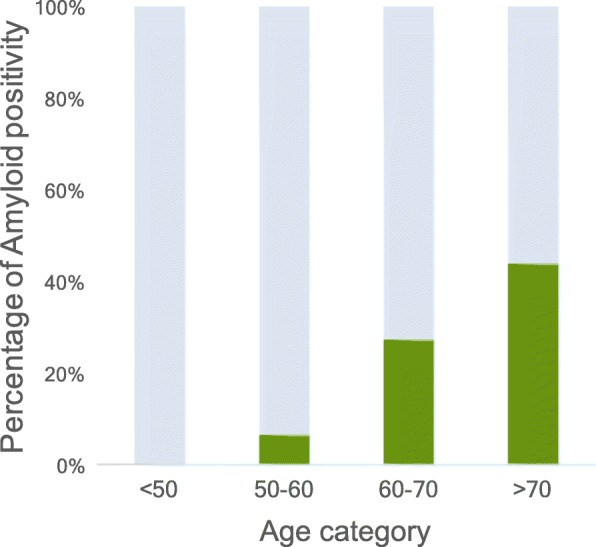
Percentage of amyloid positivity per decade. Percentage of amyloid positivity per age category in participants with available amyloid status (*n* = 114)

When we attempted to categorize participants according to the presence of preclinical AD and/or subthreshold psychiatric symptoms, we found 28 individuals (25% of 114 participants with known amyloid status, and 18% of total sample) with preclinical AD. Higher age was associated with an increased risk of preclinical AD (odds ratio 1.14 (95% CI 1.06–1.22); Fig. 3).

In the remaining sample, 58 (38%) participants reported subthreshold psychiatric symptoms on one or more questionnaires. Of these participants, 21% had subthreshold symptoms in the affective cluster, for example depressive (11%) and/or anxiety (13%) symptoms. Roughly one out of three (31%) had distress and/or somatization-related symptoms, and in 27% there was an indication of symptoms of neuroticism and/or low mastery. In addition, 8 of 28 (29%) patients in the preclinical AD category also had subthreshold psychiatric symptoms.

The largest group of SCD (*n* = 65 (43%)) had neither evidence of amyloid nor of subthreshold psychiatric symptoms (undetermined category).

Comparing these three SCD groups, participants with preclinical AD were on average older than individuals in the subthreshold psychiatry (*p* < 0.001; Table 1) and undetermined category (*p* < 0.05). Participants with preclinical AD more frequently had a family history of dementia than subthreshold psychiatry, and they were APOE e4 carriers more frequently than the other two groups (all *p* < 0.01). There were no differences in gender, education, or MRI measures between groups. Self-reported cognitive decline was higher in participants with subthreshold psychiatry than in the undetermined category, with preclinical AD in between (both *p* < 0.01). Results were similar for informant-reported cognitive decline. Reported quality of life was lower in the subthreshold psychiatry group than in the undetermined category (*p* = 0.002), with preclinical AD in between. Comparing objective cognitive performance between groups, the group with subthreshold psychiatry performed worse on the TMT-A compared to preclinical AD and undetermined groups (all *p* < 0.05). Also, subthreshold psychiatry performed worse on the TMT-B than the undetermined group (*p* < 0.05), but there were no differences in other cognitive tests, see Table 1.

### SCD-plus criteria and the risk of preclinical AD

**Fig. 4:**
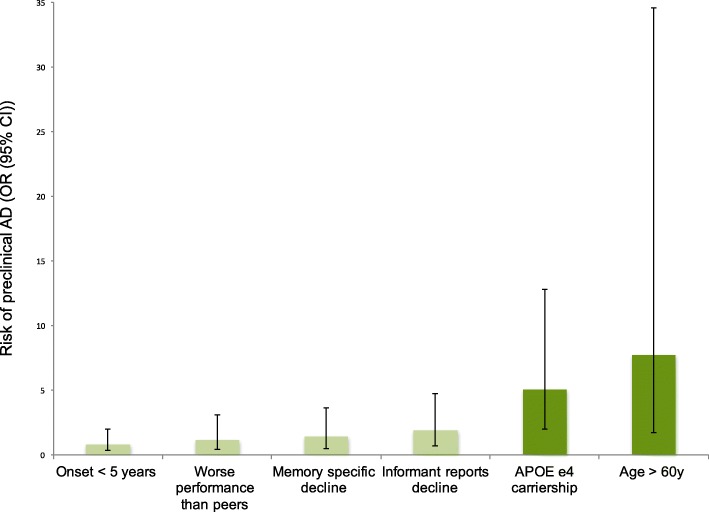
SCD-plus criteria and risk of preclinical AD. Risk of preclinical AD for each SCD-plus criterion in participants with available amyloid status (*n* = 114). AD Alzheimer’s disease, APOE apolipoprotein E (genotype), CI confidence interval, OR odds ratio

Univariate logistic regression analyses showed that SCD-plus criteria ‘age ≥ 60’ (OR 7.7 (95% CI 1.7–34.6)) and ‘APOE e4 carriership’ (OR 5.0 (2.0–12.8)) were associated with an increased risk of preclinical AD (Fig. 4), whereas ‘memory specific decline’, ‘onset of complaints within 5 years’, ‘worse performance than other of the same age’, and ‘informant reports decline’ were not (Table 3). In a multivariate stepwise model, APOE e4 carriership (OR 6.2 (1.7–22.2)) and age ≥ 60 (OR 3.8 (1.7–20.4)) remained independently associated with preclinical AD.

**Table 3:** SCD-plus criteria and the risk of preclinical AD in individuals with available amyloid status (*n* = 114)

Predictor	Data availability (*n*)	Prevalence of SCD-plus criteria^a^		Risk of preclinical AD^b^	
	in group with known amyloid status (*n* = 114)	Preclinical AD (*n* = 28)	Amyloid negative (*n* = 86)	Univariate model	Multivariate stepwise model
Memory specific decline	94	13 (59%)	37 (51%)	1.4 (0.5–3.6)	–
Onset < 5 years	111	12 (46%)	43 (51%)	0.8 (0.3–2.0)	–
Age ≥ 60 years	114	26 (93%)	54 (63%)	7.7 (1.7–34.6)	3.8 (1.7–20.4)
Experience of worse performance than others	90	13 (65%)	44 (63%)	1.1 (0.4–3.1)	–
Informant reports decline	97	15 (60%)	32 (44%)	1.9 (0.7–4.7)	–
APOE e4 carriership	110	17 (65%)	23 (27%)	5.0 (2.0–12.8)	6.2 (1.7–22.2)

*AD* Alzheimer’s disease, *APOE* apolipoprotein E (genotype), *SCD* subjective cognitive decline

^a^Prevalence of each SCD-plus criterion in individuals with and without preclinical AD, presented as *n* (%), within cases with amyloid status available (*n* = 114)

^b^Risk of preclinical AD separately (univariate models) for each SCD-plus criterion and independent predictors of preclinical AD in a multivariate stepwise model in SCIENCe participants with available amyloid status (*n* = 114), presented as odds ratio (95% confidence interval)

## Discussion

The SCIENCe project aims to investigate factors potentially related to SCD. Cross-sectional evaluation of the first 151 cognitively normal participants with SCD revealed a heterogeneous group, with preclinical AD in one fifth to one quarter of participants, and subthreshold psychiatric symptoms in more than one third of participants, while the largest group of participants did not have evidence of either. We found that higher report of SCD was associated with lower quality of life, and also with worse cognitive performance. Finally, the SCD-plus criteria age ≥ 60 and APOE e4 carriership were associated with an increased risk of preclinical AD, defined by amyloid positivity on either PET or in CSF.

We measured the degree of subjective complaints with the short SCF questionnaire and used the CCI for more in-depth evaluation [13, 20]. Almost all participants reported cognitive decline, which seems substantially higher than in the general population [36], and could be a reflection of our cohort with individuals actively seeking medical evaluation in a memory clinic because of these cognitive complaints. A small minority of 3% did not report any complaints, potentially explained by the fact that participants filled in the questionnaires after a thorough memory clinic evaluation, with reassurance of normal cognitive functioning.

We found that higher report of cognitive complaints was associated with worse quality of life, suggesting that subjective complaints have a negative effect on a general feeling of wellbeing. On the other hand, we cannot exclude reverse causality, as worse quality of life may also affect the subjective appreciation of one’s (cognitive) abilities [37]. Furthermore, we found that higher report of cognitive complaints on the CCI (both self and informant) was associated with worse objective cognitive performance in our cognitive normal sample with SCD, which is in line with the literature on the CCI and objective performance [38]. Although self-report of SCD has been associated with future cognitive decline [5, 8], and also has been suggested to be more sensitive for subtle decline than informant report in the very earliest stages of cognitive decline, earlier cross-sectional associations have not been consistent [11, 39–41]. This could be a result of the use of different SCD measures [42]. Indeed, we found no significant associations between objective cognition and SCF, which measures cognitive complaints over a shorter period of time and consists of four questions only, in contrast to the observed associations with the CCI.

Cognitive complaints in cognitively normal individuals were previously found to have a broad range of associated symptoms, varying from distress to affective disorders, systemic illnesses, and preclinical AD [11, 12, 15, 16, 39]. In the current paper we evaluated the prevalence of preclinical AD and subthreshold psychiatric features as potential factors associated with the occurrence of SCD [4]. We observed that 25% of participants with available amyloid status had preclinical AD, and amyloid positivity increased with age. Although we did not make a formal comparison, percentages of amyloid positivity per decade seem somewhat higher in our cohort than in individuals with SCD in a recent large meta-analysis investigating amyloid prevalence in the nondemented elderly [43].

In our sample, 38% of participants experienced subthreshold psychiatric symptoms on one or more domains. These symptoms were labeled subthreshold since individuals with a clear psychiatric diagnosis, such as major depression, were not included. The group with subthreshold psychiatric symptoms reported more cognitive complaints than the group with preclinical AD. We evaluated six psychiatric features which have been previously associated with cognitive complaints in individuals with SCD, and might provide an alternative explanation for the subjective experience of decline [15, 16, 39, 41, 44]. On the other hand, several of these psychiatric features, such as depressive symptoms, anxiety, neuroticism, and distress, have also been associated with preclinical AD [45–51], and, indeed, we also saw the co-occurrence of preclinical AD and subthreshold psychiatric symptoms in 8 of 28 cases. We are currently following all participants to study clinical progression in these different groups.

For 43% of the remaining SCIENCe participants, we found neither preclinical AD nor subthreshold psychiatry. Individuals in the undetermined category had fewer cognitive complaints than the other two categories, both reported by themselves and by the informant. Nonetheless, each of these patients was referred to the memory clinic for evaluation of complaints. In the undetermined category we found a higher prevalence of family history of dementia than in the subthreshold psychiatry category, similar to preclinical AD. Perhaps anxiousness related to family history of dementia, rather than the actual experience of cognitive decline, could be a reason to visit the memory clinic for evaluation [52].

To facilitate harmonization of SCD research, the international SCD Working Group (SCD-I) has published a conceptual framework on SCD research, which included the SCD-plus criteria as determinants of preclinical AD [4]. This is the first time the SCD-plus criteria were comprehensively evaluated in a clinical setting. We found that the SCD-plus criteria age ≥ 60 and APOE e4 carriership were associated with an increased risk of preclinical AD, which is in line with the literature [11, 43, 53]. The four other SCD-plus criteria we evaluated were not associated with preclinical AD in our cohort. There was a trend for an increased risk of preclinical AD when the informant reported significant decline, but results were not significant. The lack of association between informant report and preclinical AD is in contrast with a previous study showing an association between these factors [54]. This contrast could possibly be explained by differences in informant report measurement methods, as well as differences in sample size between the previous study and ours. Since informant report seems to be a better predictor of future cognitive decline than patient report [7, 10, 55], future longitudinal evaluation of SCIENCe participants and extension of sample size may reveal further relations. Furthermore, the criteria ‘worse performance than others of the same age’ and ‘memory specific decline’ were not associated with an increased risk of preclinical AD, which is in contrast to previous studies indicating both concepts to be associated with preclinical AD [11, 56]. We used questions from the CCI and SCF to assess these topics (respectively *feeling of worse performance than others* (yes/no) and *how do you evaluate your memory function compared to 1 year ago* (stable/decline)). For these two SCD-plus criteria, differences between results may be caused by methodological variation in SCD measurements, which are known to result in great variation between studies [42]. Criterion ‘onset of symptoms within 5 years’ did not alter the risk of preclinical AD in our cohort. To our knowledge this is the first study evaluating the association between onset of symptoms within 5 years and preclinical AD, whereas others evaluated the risk of future cognitive decline in relation to onset of symptoms, without taking into account preclinical AD [57–59].

Limitations of the study include the availability of amyloid status in the cohort for 114 of 151 participants. Because of the availability of amyloid status, participants that are now classified in the subthreshold or undetermined category may have preclinical AD of which we are unaware, since we hierarchically first included participants in the preclinical AD group, followed by categorization of the remaining participants (amyloid status negative or unknown) in the other two groups. Strengths of the study include the highly standardized assessment of a broad range of factors potentially related to SCD, including various biomarkers, as well as repeated collection of blood and CSF for biobanking to be able to evaluate biomarkers longitudinally.

In the light of a disease evolving over decades, longitudinal evaluation seems necessary to assess if, and when, those with and without preclinical AD eventually show progression to MCI or dementia. In SCIENCe we aim to evaluate which factors predict progression, but also which factors are protective of future decline. Discriminating preclinical AD from the ‘worried well’ seems especially important as anti-amyloid therapies targeting early stages of AD appear a realistic possibility in the nearby future [60]. Furthermore, assessment of factors other than preclinical AD contributing to SCD may be of importance, since also nonpharmacological interventions seem to be of added value in individuals with SCD [61].

## Conclusions

In summary, this first cross-sectional evaluation of SCIENCe participants revealed that SCD is a heterogeneous group, with subthreshold psychiatric features alongside preclinical AD. We found that subjective report of decline was associated with objective measures. Furthermore, we found a number of SCD-plus criteria to be associated with preclinical AD. Further inclusion and follow-up will address important questions related to SCD.

## Additional file

Additional file 1: **Table S1.** Standardized tests and questionnaires used in the SCIENCe project. (PDF 323 kb)

## Abbreviations

Aβ: Amyloid-beta
AD: Alzheimer’s disease
APOE: Apolipoprotein E (genotype)
CCI: Cognitive Change Index
CSF: Cerebrospinal fluid
MCI: Mild cognitive impairment
MMSE: Mini-Mental State Examination
PET: Positron emission tomography
RAVLT: Rey Auditory Verbal Learning Test
sβ: Standardized beta
SCD: Subjective cognitive decline
SCF: Subjective Cognitive Functioning
SCIENCe: Subjective Cognitive Impairment Cohort
TMT: Trail Making Test
