# Supplementary material for: Subjective Cognitive Impairment Cohort (SCIENCe): study design and first results
Source: Alzheimers Res Ther. 2018 Aug 7;10:76. doi: 10.1186/s13195-018-0390-y (PMC6080529; doi:10.1186/s13195-018-0390-y)
Supplement: Supplementary file 1 — Table S1. Standardized tests and questionnaires used in the SCIENCe project. (PDF 323 kb) [file 13195_2018_390_MOESM1_ESM.pdf]

SUPPLEMENT manuscript ‘Subjective Cognitive Impairment Cohort (SCIENCe): study design and first results’

Rosalinde E.R. Slot, MD

table S1. Standardized tests and questionnaires used in the SCIENCe project

Category	Domain	Name of test or questionnaire	Cut-off
Clinical evaluation	Anamnesis	Anamnesis, evaluation of complaints<br>Medical history<br>Family history<br>Alcohol intake, smoking, drugs	
	Physical measurements	Weight, height, waist<br>Blood pressure	
Subjective cognitive decline	Self-perceived decline	Cognitive change index – self-report, functioning compared to 5 years ago [1]	< 0
		Subjective cognitive functioning, change over 1 year [2]	≥ 16
	Informant report	Cognitive change index – informant report [1]	≥ 16
Quality of life		EuroQol, visual analogue scale [3]	
Mental health questionnaires	Depressive symptoms	Center for Epidemiologic Studies Depression Scale (CES-D) [4]	≥ 16
	Anxiety	Hospital anxiety and depression scale (HADS) – anxiety subscale [5]	≥8
	Distress	Four Dimensional Symptom Questionnaire – distress subscale [6]	≥10
	Somatization	Four Dimensional Symptom Questionnaire – somatization subscale [6]	≥10
	Neuroticism	Dutch Personality Inventory – Neuroticism scale [7]	80th percentile current report
	Mastery	Pearlin Mastery psychological coping scale [8]	80th percentile current report
Neuropsychological tests	Global cognition	Mini-mental state examination [9,10]	
	Memory	Visual association task (VAT) A&B [11]<br>Dutch version of the Rey Auditory Verbal Learning Test (RAVLT) – direct recall; delayed recall and cued recall after 20 min [12,13]<br>Visual Reproduction I&II (Wechsler Memory Scale (WMS) IV) [14]<br>Story immediate and delayed recall (Rivermead Behavioral Memory Test (RBMT) III, Dutch version) [15,16]<br>Rey Complex Figure delayed recall (3 min) [17]	
	Attention	Digit span forward [18]<br>Trail Making Test (TMT) A [19]<br>Stroop word naming (I) [16,20]<br>Stroop colour naming (II) [16,20]	
	Executive functioning	Trail Making Test (TMT) B [19]<br>Digit span backwards [18]<br>Stroop colour-word test [16,20]<br>Frontal Assessment Battery (FAB) [21]<br>Letter Fluency Test (version D-A-T) [22]	
	Language	Category fluency animals [16]	

		Boston Naming Test (30 items) [23–25]
		Visual association test – 'naming' [11]
	Visuo-spatial functioning	Fragmented letters (VOSP: Visual Objective and Space Perception) [26]
		Number location (VOSP) [26]
		Dot Counting (VOSP) [26]
		Rey Complex Figure Copy task [17]
Activities of daily living	IADL	Amsterdam IADL questionnaire [27]
Lifestyle questionnaires	Dietary intake	EETscore [28]
	Physical activity	Physical Activity Scale for the Elderly [29]
	Cognitive activity	Cognitive Activity Scale [30]

REFERENCES

[1] Saykin AJ, Wishart HA, Rabin LA, Santulli RB, Flashman LA, West JD, McHugh TL, Mamourian AC (2006) Older adults with cognitive complaints show brain atrophy similar to that of amnestic MCI. *Neurology* **67**, 834–842.

[2] Aalten P, Ramakers IH, Biessels GJ, de Deyn PP, Koek HL, OldeRikkert MG, Oleksik AM, Richard E, Smits LL, van Swieten JC, Teune LK, van der Lugt A, Barkhof F, Teunissen CE, Rozendaal N, Verhey FR, van der Flier WM (2014) The Dutch Parelsnoer Institute - Neurodegenerative diseases; methods, design and baseline results. *BMC Neurol.* **14**, 1–8.

[3] Brooks R, The EuroQol Group (1996) EuroQol : the current state of play *. *Health Policy (New. York).* **37**, 53–72.

[4] Radloff LS (1977) The CES-D Scale: A Self-Report Depression Scale for Research in the General Population. *Appl. Psychol. Meas.* **1**, 385–401.

[5] Bjelland I, Dahl A a, Haug TT, Neckelmann D (2002) The validity of the Hospital Anxiety and Depression Scale. *J. Psychosom. Res.* **52**, 69–77.

[6] Terluin B, Rhenen W Van, Schaufeli WB, De Haan M (2004) The four-dimensional symptom questionnaire (4DSQ): measuring distress and other mental health problems in a working population. *Work Stress* **18**, 187–207.

[7] Luteijn F, Staren J, van Dijk H (1985) *Handleiding bij de Nederlandse Persoonlijkheids Vragenlijst (Manual for the Dutch Personality Questionnaire)*, Swets & Zeitlinger, Lisse, The Netherlands.

[8] Pearlin LI, Schooler C (1978) The structure of coping. *J. Health Soc. Behav.* **19**, 2–21.

[9] Folstein M (1975) "Mini-mental state". A practical method for grading the cognitive state of patients for the clinician. *J. Psychiatr. Res.* **12**, 189–198.

[10] Kok RM, Verhey FRJ, Schmand B (2004) Meetinstrumenten bij cognitieve stoornissen. *Tijdschr. Psychiatr.* **46**, 665–670.

[11] Lindeboom J, Schmand B, Tulner L, Walstra G, Jonker C (2002) Visual association test to detect early dementia of the Alzheimer type. *J. Neurol. Neurosurg. & Psychiatry* **73**, 126 LP-133.

[12] Rey A (1964) *L'examen clinique en psychologi (The clinical examination in psychology), 2nd ed.*, Presses Universitaries De France, Paris.

[13] Saan RJ, Deelman BG (1986) *De 15-Woorden Test A en B. (Een voorlopige handleiding).*, , Groningen.

[14] Bowden SC, Carstairs JR, Shores EA (1999) Confirmatory factor analysis of combined Wechsler Adult Intelligence Scale—Revised and Wechsler Memory Scale—Revised scores in a healthy community sample. *Psychol. Assess.* **11**, 339–344.

[15] Wilson B, Greenfield E, Clare L, Baddeley A, Cockburn J, Watson P, Nannery R (2003) *Rivermead behavioural memory test – third edition*, , Toronto.

[16] Schmand B, Houx P, de Koning I (2003) *Normen voor Stroop kleur-woord tests, Trail Making test, en Story Recall van de Rivermead Behavioural Memory Test, Uitgave onder auspiciën van de sectie Neuropsychologie van het Nederlands Instituut van Psychologen Amsterdam*, , Amsterdam.

[17] Osterrieth PA (1944) Filetest de copie d'une figure complex: Contribution a l'etude de la perception et de la memoire [The test of copying a complex figure: A contribution to the study of perception and memory]. *Arch. Psychol. (Geneve).* **30**, 286–356.

[18] Lindeboom J, Matto D (1994) Cijferreeksen en Knoxx blokken als concentratietests voor ouderen. *Digit Ser. Knox cubes as Conc. tests Elder. Subj. [in Dutch]* **25**, 63–68.

[19] Reitan RM (1958) Validity of the Trail making test as indicator of organic brain damage. *Percept. Mot. Skills* **8**, 271–276.

[20] Bouma JM, Mulder J, Altena E, Schmand B, Lindeboom J (2012) Stroop Kleur-Woord Test (Stroop KWT). In *Handboek neuropsychologische diagnostiek* Pearson, Amsterdam, pp. 475–509.

[21] Dubois B, Slachevsky A, Litvan I, Pillon B (2000) The FAB: A frontal assessment battery at bedside . *Neurol.* **55**, 1621–1626.

[22] Schmand B, Groenink SC, van den Dungen M (2008) [Letter fluency: psychometric properties and Dutch normative data]. *Tijdschr. Gerontol. Geriatr.* **39**, 64–76.

[23] Kaplan E, Goodglass H, Weintraub S (1976) *Boston Naming Test, Experimental edition*, , Boston.

[24] Van Loon-Vervoorn WA, Stumpel HJ, De Vries LA (1995) *De Boston BenoemingsTaak – Een test voor woordvinding bij afasie*, , Utrecht.

[25] Schoonk-Lammers MJ, Heesbeen IME, Van Loon-Vervoorn WA (2004) Uitbreiding normering van de Boston BenoemingsTest. De invloed van sekse, leeftijd en opleiding op de 104 benoemvaardigheid bij personen met niet-aangeboren hersenletsel. *Logop. en Foniatr.* **11**, 752–760.

[26] Herrera-Guzman I, Pena-Casanova J, Lara JP, Gudayol-Ferre E, Bohm P (2004) Influence of age, sex, and education on the Visual Object and Space Perception Battery (VOSP) in a healthy normal elderly population. *Clin. Neuropsychol.* **18**, 385–394.

[27] Sikkes SAM, De Lange - De Klerk ESM, Pijnenburg YAL, Knol DL, Uitdehaag BMJ, Scheltens P (2011) Validation of the Amsterdam IADL questionnaire®, a new tool to measure instrumental activities of daily living. *Alzheimer's Dement.* **7**, S4–S5.

[28] Lee L Van, Feskens EJM, Meijboom S, Hooft Van Huysduynen EJC, Van't Veer P, De Vries JHM, Geelen A (2016) Evaluation of a screener to assess diet quality in the Netherlands. *Br. J. Nutr.* 517–526.

[29] Geffken DF, Cushman M, Burke GL, Polak JF, Sakkinen PA, Tracy RP (2001) Association between Physical Activity and Markers of Inflammation in a Healthy Elderly Population. *Am. J. Epidemiol.* **153**, 242–250.

[30] Wilson RS, Barnes LL, Bennett DA (2003) Assessment of Lifetime Participation in Cognitively Stimulating Activities. *J. Clin. Exp. Neuropsychol.* **25**, 634–642.
